# The Role of Radiotherapy and Chemotherapy in the Treatment of Primary Adult High Grade Gliomas: Assessment of Patients for These Treatment Approaches and the Common Immediate Side Effects

**DOI:** 10.5402/2012/902178

**Published:** 2012-12-11

**Authors:** E. E. Philip-Ephraim, K. I. Eyong, U. E. Williams, R. P. Ephraim

**Affiliations:** ^1^Department of Internal Medicine, College of Medical Sciences, University of Calabar, PMB 1278, Nigeria; ^2^Department of Paediatrics, College of Medical Sciences, University of Calabar, PMB 1278, Nigeria

## Abstract

Gliomas are the commonest primary brain tumours in adults. They are usually classified and graded according to the criteria by the World Health Organisation. High-grade gliomas are the most malignant primary brain tumours. Conventional therapies include surgery, radiotherapy, and chemotherapy. The tumours often demonstrate high levels of resistance to these conventional therapies, and in spite of treatment advances the prognosis remains poor.

## 1. Introduction

Primary brain tumours are tumours that arise within the brain tissue and the environs while secondary tumours originate from elsewhere in the body and spread to the brain [1]. The main functional cell of the brain is called the neuron, while glial cells make up the supporting structures. These glial cells are of different types, namely, the oligodendrocytes which make myelin, astrocytes which take part in neurotransmission and neuronal metabolism, and the ependyma cells which line the ventricles and central canal of the spinal cord. Tumours arising from glial cells are called gliomas. These tumours are the most frequent primary tumours of the central nervous system [2, 3].

The World Health Organisation (WHO) classifies astrocytomas into four grades, depending on the microscopic pattern of the tumour, which include increased cellularity, nuclear atypia, mitosis, and vascular proliferation/necrosis. The different characteristics demonstrate the invasiveness and rate of growth of the various grades.

WHO Grade I (Pilocytic astrocytoma) is that which does not demonstrate any of the microscopic patterns.

WHO Grade II (diffuse astrocytoma) is characterized by only atypia.

WHO Grade III (anaplastic astrocytoma) shows both atypia and mitosis. 

WHO Grade IV (glioblastoma multiforme) shows areas of necrosis and/or vascular proliferation [1, 4].

The tumours in WHO Grades I and II are referred to as low-grade gliomas while those in grades III and IV are called *high*-*grade gliomas* (HGG). By far, the HGG are the commonest primary brain tumours in adults [5, 6]. They can develop de novo or result from a progression of the low-grade gliomas [7–9]. Seventy percent of the 22,500 new cases of malignant primary brain tumours diagnosed in adults in the United States are due to HGG exhibiting an incidence of 5 per 100,000/per year [4, 10]. The most common type of HGG is glioblastoma accounting for 80% of the malignant gliomas while the Oligodendrogliomas constitute about 20% of glial tumours [11]. 

Males are more frequently affected than females with a ratio of 3 : 2 [3, 11]. A possible protective effect of the female hormones has been proposed as a cause since the difference is more noticeable among women of child-bearing age [3]. The HGG can occur in any part of the brain but normally involve the cerebral hemispheres [11]. Glioblastoma affects elderly people with a mean age at diagnosis of 64 years while anaplastic astrocytoma affects younger patients of about 45 years [4, 11]. The codeletion of 1*p*/19*q* in anaplastic Oligodendrogliomas correlates with better survival and chemosensitivity. Poor survival in anaplastic astrocytoma is linked to loss of phosphate and tensin homolog *(PTEN)* tumour suppressor gene. Also poor survival in glioblastoma patients is linked to epidermal growth factor receptor overexpression, p53 expression, and *MDM2* overexpression [3]. The HGG are highly aggressive malignant tumours with very poor prognosis. The mean survival for anaplastic astrocytoma is 3–5 years and much shorter, less than 1 year, for glioblastoma patients [12]. Prognostic features include performance status, age, and histologic characteristics [13].

Headache is the initial symptom in about 30% of cases. The headaches, generally dull in character, are difficult to differentiate from tension headaches. Symptoms of increased intracranial pressure may be present. Seizures can also occur but are less common in glioblastomas probably due to decreased life expectancy in these patients [14]. Cognitive impairment can occur in up to 40–60% of patients with glioblastoma. Personality and mood changes do also occur. A focal neurological deficit with weakness of one side of body has been reported [4, 11, 14]. 

## 2. The Role of Radiotherapy

The treatment of HGG entails different modalities including surgery followed by radiotherapy and chemotherapy. This multimodality approach is still regarded as the mainstay of treatment [13, 15, 16]. Occasionally, when complete resection is impossible, radiotherapy can then be the initial treatment. A thorough treatment planning is therefore necessary for a favourable outcome in radiotherapy [17]. HGG is noted for local recurrence and because of this studies have led to ways of maximizing radiation doses locally to enhance control.

### 2.1. Conventional Radiotherapy

Various studies carried out in the 1960's to compare the outcome of those receiving radiotherapy (RT) with nonrecipient reported better prognosis in the group that received RT [18, 19]. Despite this, debate regarding the best way to deliver RT remains. A delay in commencement of RT after surgery has been associated with poor prognosis [20].

#### 2.1.1. Dose

Several studies have been carried out comparing radiation dose of 45 Gy 20 fractions over 4 weeks to 60 Gy 30 fractions over 6 weeks as relates to the outcome of HGG. A higher dose of 60 Gy was found to correlate with a better prognosis as relates to overall survival [10, 21]. In another trial, a higher dose of 70 Gy showed no further improvement in survival compared with those that received 60 Gy dose, proving that a dose higher than 60 Gy had no extra advantage [18, 19] but rather can be associated with increased toxicity [10].

#### 2.1.2. Radiation Volume

 The 60 Gy whole brain RT utilized in the 1960's and 1970's in treatment of HGG is now restricted to patients with multifocal gliomas. Local brain irradiation with margins of 2 cm around the tumour mass is being preferred [18]. 

This is because of better tumour localization by CT and MRI in this era and also due to reports of tumour recurrence at the primary site in over 90% of cases [10]. Local brain irradiation has the beneficial effect of reducing the volume of brain tissue irradiated thereby reducing the toxic effect of the radiation without affecting tumour control [19]. Few studies have investigated the volume of radiotherapy delivery. No statistical difference was found in survival rate and morbidity between the groups that received whole brain RT and local brain RT [22, 23] whereas performance status was found to be better in recipients of localized field RT [23].

### 2.2. Altered Fractionation

- Hyperfractionated radiotherapy involves the giving of a higher total dose than the conventional irradiation using multiple small-sized fractions over the same overall treatment period. The benefit of this was in allowing a higher dose to be delivered without much morbidity due to lower fraction size. Almost all the studies carried out showed no advantage in the use of hyperfractionated over conventional RT [18, 19, 24].
- Accelerated hyper-fractionated radiotherapy requires the delivery of multiple fractions in the standard dose over a shortened treatment time. Studies done neither showed increase in toxicity nor improvement in survival rate over the hyperfractionation or the conventional RT [10, 18, 19, 25].
- Hypofractionation, here, larger than normal-sized radiation fractions with a reduced total radiation dose and a shortened overall treatment time is employed. This regimen is used not only in older age but also in patients with poor prognosis and unresectable tumours [19]. Hypofractionation showed glioblastoma patients to have a favourable response in terms of survival rate over the conventional fractionation [26] while other studies concluded that hypofractionation had similar results and tolerance comparable with conventional fractionation [27, 28].

### 2.3. Brachytherapy

This employs the placement of radiation isotopes directly inside and around the tumour. This method significantly increases the delivery of a higher tumour dose radiation without much risk to the surrounding brain tissue. Beneficial effect is seen mostly in patients with good karnofsky score and small unifocal tumours. Studies carried out showed encouraging results in the median survival of these patients [29, 30] although other studies could not confirm a statistically survival advantage for brachytherapy [18].

### 2.4. Radiosurgery

This entails the delivery of a stereotactically radiation beam in single fraction to focus on the tumour site. 

### 2.5. Other Modalities

This includes the use of hyperthermia, particle therapy, and radiosensitizers.

## 3. The Role of Chemotherapy

The medical treatment of HGG has been a challenge because the drugs must be able to have not only potent and accurate cytotoxic effect but also be able to penetrate the blood brain barrier (BBB) and overcome any mechanism of drug resistance. Chemotherapy is used concomitantly or as an adjuvant to RT. The inclusion of chemotherapy in HGG is found to improve survival significantly [31].

### 3.1. Conventional Chemotherapy

- *Nitrosoureas. *These are alkylating agents and are lipid-soluble which allows them to overcome the BBB successfully. Examples include carmustine, lomustine, and nimustine. They have been used orally, intra-arterially, and as wafers in the treatment of HGG. Nitrosoureas have been studied in different contexts and found to demonstrate prolongation in survival as relates to extension of tumour progression time [32].
- *Procarbazine, lomustine, and vincristine (PCV).* In an attempt to improve the efficacy of the nitrosoureas, the combination therapy was utilized. Although beneficial effect of PCV as regards prolongation of both survival and tumour progression time amongst anaplastic glioma patients was established [33], nonetheless no demonstrable difference in survival advantage to PCV over carmustine was found in a retrospective study [34].
- *Temozolomide (TMZ).* This is a newer alkylating agent with excellent bioavailability. The O^6^- methylguanine-DNA methyl transferase repair protein (*MGMT)* has been implicated in the resistance to the alkylating cytotoxic drugs. The outcome of methylation of the *MGMT *promoter is inactivation of the gene which is said to be responsible for the increased sensitivity to treatment. The benefit of Temozolomide in glioblastoma has been linked to the methylation of the *MGMT* promoter [35]. TMZ is usually given orally at a dose of 200 mg/m^2^ daily for 5 days and then repeated after 28 days. Trials with TMZ have been carried out in various contexts. These studies have consistently demonstrated clear benefit creditable to TMZ as regards progression free survival, increase in quality of life, and increase in 2 year survival [36–38]. In one of the studies, glioblastoma patients that were diagnosed newly were given TMZ at a dose of 75 mg/m^2^ daily for 6 weeks alongside with radiotherapy followed by TMZ alone at a dose of 200 mg/m^2^/day for 6 cycles. The total number of patients studied was sixty four. This regimen was well accepted and led to a “median survival time of 16 months, with 1- and 2-year survival rates of 58% and 31%, respectively” [39]. Because of these outcomes, concurrent use of TMZ and RT is now regarded as the standard treatment for newly diagnosed glioblastoma patients [31].
- *Platinum salts.* These are also alkylating agents and are used mainly in treating recurrent HGG. Two examples of this class are carboplatin and cisplatin. Carboplatin was shown to be effective against recurrent HGG as 50% of the assessed patients demonstrated increase in their median survival time of 38 weeks while median time to progression of the tumour was 19 weeks [40].
- *Topoisomerase 1 and 11 Inhibitors.* Etoposide is the prototype of type 11 and exerts its action by inhibiting DNA topoisomerase 11 enzymes [41]. The potent synergistic effect of the platins and etoposide has led to the conduction of various trials. In one of the trials, the combination of carboplatin and etoposide showed a median survival time of 10 months when 30 patients were treated [42]. Examples of type-1 inhibitors include Irinotecan and topotecan. Despite the high toxicity displayed by these drugs, the median survival time of 15 months over the control of 11 months was encouraging when topotecan was used concomitantly in an open-labelled study [43].
- *Other Cytotoxics.* These include taxane derivatives and anthracyclines. Some investigators found modest beneficial effect in the use of taxane in HGG [44, 45].

### 3.2. Biological Modifiers

These are agents with cytostatic action. Different types have been tried in the treatment of HGG. They include Vitamin A and D derivatives, matrix metalloprotease inhibitors, angiogenesis inhibitors, and signal transduction inhibitors like tamoxifen. Various studies conducted showed successes in some while others failed to prove any benefit as regards progression and survival time when compared with placebos in glioblastoma patients [31].

### 3.3. Intra-Arterial Chemotherapy

The aim of this mode of therapy is to deliver a sufficient dose of the drug into the tumour site. This regimen has been used in various trials for the management of both recurrent as well as newly diagnosed HGG [46]. Intracarotid and intracerebral studies of cisplatin and carmustine, respectively, as single agents have been encouraging as regards disease stabilization and response rates [47, 48]. Although intra-arterial administration of drugs has been successful, other drugs like etoposide have produced discouraging outcomes for primary malignant brain tumours [49].

### 3.4. Autologous Bone Marrow Rescue

In an effort to improve the long-term survival of patients with HGG, the use of high-dose chemotherapy followed by autologous bone marrow rescue has been studied in various trials. Even though remarkable results were obtained with the use of high dose BCNU, the toxic effects produced were enormous [50, 51]. 

### 3.5. Polymer-Drug Delivery

This method enhances a sustained local drug delivery and exposure, circumvents the BBB and the unnecessary systemic toxic effects. In a multicentre randomized placebo-controlled trial, the authors were able to demonstrate the effectiveness of carmustine in prolonging survival time in patients with recurrent gliomas [52].

### 3.6. Intratumoral Chemotherapy

Here treatment is delivered directly into the tumour or tumour bed after resection. Advantages of this mode of therapy include maximal concentration of the drug intratumorally, by-pass of BBB, and reduction in unwanted systemic toxic effects. Success has been reported with this mode of treatment [53].

#### 3.6.1. Blood Brain Barrier

The BBB lowers the efficiency of cytotoxic drugs. In an attempt to disrupt this barrier, intra-arterial Mannitol has been used to increase the uptake of the cytotoxic drugs [54].

## 4. Assessment of Patients for These Treatment Approaches

The assessment of patients with HGG requires a multidisciplinary approach involving the neurologists, neurosurgeons, neuropathologists, and the neuropsychologists/neuropsychiatrists.

### 4.1. The Neurologist Assessment

A complete history of the patient will be taken. A thorough general medical and neurological examination can suggest a diagnosis of gliomas. Disease conditions like hypertension and the Ischaemic heart diseases will have to be excluded. Other comorbidites like asthma, kidney failure, bleeding disorders, diabetes mellitus and hepatitis should be excluded. Investigations should include a full blood count, clotting profile, blood glucose, serum electrolyte, urea, creatinine and grouping, and cross matching [14].

### 4.2. The Neuropsychologist Assessment

This assessment is vital as the initial symptom can be cognitive impairment which can progress during and even after therapy. Assessment of cognitive function using Karnofski Performance Score is usually carried out. In spite of its limitations, it is still used in the evaluation of quality of life [55].

### 4.3. The Neuropsychiatry Assessment

Patients with primary brain tumours tend to have depression, apathy, psychosis, and personality changes. Counseling of the patient and usage of different modalities of treatment to stabilize the patient makes the neuropschiatrist indispensable [56].

### 4.4. The Neuropathologist

The cornerstone for confirmation of HGG is by tissue biopsy. The biopsied sample can then be examined histologically, not only to confirm the diagnosis but also to characterize the different grades of gliomas. For precision, the pathologic diagnosis is interpreted in relation to the clinical history by the neurologist as well as findings of the neurosurgeons and neuroradiologist [57]. Immunohistochemistry studies can also be done. Thus GFAP (Glial fibrillary acidic protein) seen almost exclusively in HGG can strengthen the diagnosis. Genetic testing that can be done include *PTEN, TP53, MDM2, EGFR* overexpression, and *1p/19q* codeletions [58]. These tests have prognostic values in chemotherapy.

### 4.5. The Neuroradiologist Assessment

Advancement in neuroimaging techniques has significantly improved the assessment of HGG. Magnetic Resonance Imaging (MRI) and Computed Tomography (CT) in addition to playing investigative roles in the diagnosis of HGG are used in planning therapy, and a followup in evaluating the success of the treatment modalities [59]. A nonhomogenous enhancement of the mass is often seen with MRI or CT. Functional MRI can be used in determining the location of speech and motor areas in relation to the tumour prior to surgery [4]. The use of Positron Emission tomography using FDG has also been used in differentiating glioblastoma from anaplastic astrocytoma because of regions of striking hypometabolism signifying necrosis [60]. Single-Photon Emission CT imaging has also been used in detecting recurrent gliomas. Other imaging techniques used include Proton magnetic resonance spectroscopy which distinguishes metabolite levels, contrast enhanced MRI which assesses permeability of blood vessels and perfusion imaging to assess cerebral blood volume [4].

### 4.6. The Neurosurgeon Assessment

A biopsy of the tumour is usually undertaken by the surgeon. Different methods are used and include needle biopsy, stereotactic biopsy, or free hand biopsy but all are guided by imaging techniques [61].

### 4.7. Informed Consent Will Have to Be Given by The Patient

#### 4.7.1. Common Immediate Side Effects

These are side effects that occur hours to days of commencing therapy.

**Radiotherapy**

The common immediate side effects here are usually mild and include hair loss, scalp redness, weakness, headache, fever, nausea, and vomiting [62].

**Chemotherapy**

Most of the cytotoxic drugs cause gastrointestinal effects like nausea, vomiting, loss of appetite, and diarrhea. Headache, dizziness, and fatigue can also occur. Local irritation can occur at IV infusion site of carmustine. Intracarotid carmustine can cause vision loss. Intra-arterial administration of nitrosoureas has also been associated with acute neurotoxicity like seizures and confusion [46, 63].

## 5. Conclusion

High-grade glioma remains the commonest primary brain neoplasm with a grave outcome. It has been widely studied in various clinical trials but in spite of the different aggressive approaches to therapy ranging from extensive resective surgery to adjuvant radiotherapy and concomitant/adjuvant use of single to combination chemotherapy, the prognosis still remains poor. Recent advances in Neuro-imaging and newer cytotoxics have brought some improvement in survival. A more extensive scientific knowledge might help in unraveling the “mysteries” of HGG. Cure for now is elusive, however all hope is not lost. We look forward to a better tomorrow as the clinical trials of various modalities of treatment continue.
